# Reconstitution of nuclear envelope subdomain formation on mitotic chromosomes in semi-intact cells

**DOI:** 10.1247/csf.24003

**Published:** 2024-06-04

**Authors:** Tomoko Funakoshi, Naoko Imamoto

**Affiliations:** 1 Cellular Dynamics Laboratory, RIKEN Cluster for Pioneering Research, 2-1 Hirosawa, Wako, Saitama 351-0198, Japan; 2 Graduate School of Medical Safety Management, Jikei University of Health Care Sciences, 1-2-8 Miyahara, Yodogawa-ku, Osaka 532-0003, Japan

**Keywords:** nuclear envelope reassembly, inner nuclear membrane protein, nuclear pore complex, semi-intact cell, *in vitro* reconstitution

## Abstract

In metazoans, the nuclear envelope (NE) disassembles during the prophase and reassembles around segregated chromatids during the telophase. The process of NE formation has been extensively studied using live-cell imaging. At the early step of NE reassembly in human cells, specific pattern-like localization of inner nuclear membrane (INM) proteins, connected to the nuclear pore complex (NPC), was observed in the so-called “core” region and “noncore” region on telophase chromosomes, which corresponded to the “pore-free” region and the “pore-rich” region, respectively, in the early G1 interphase nucleus. We refer to these phenomena as NE subdomain formation. To biochemically investigate this process, we aimed to develop an *in vitro* NE reconstitution system using digitonin-permeabilized semi-intact mitotic human cells coexpressing two INM proteins, emerin and lamin B receptor, which were labeled with fluorescent proteins. The targeting and accumulation of INM proteins to chromosomes before and after anaphase onset in semi-intact cells were observed using time-lapse imaging. Our *in vitro* NE reconstitution system recapitulated the formation of the NE subdomain, as in living cells, although chromosome segregation and cytokinesis were not observed. This *in vitro* NE reconstitution required the addition of a mitotic cytosolic fraction supplemented with a cyclin-dependent kinase inhibitor and energy sources. The cytoplasmic soluble factor(s) dependency of INM protein targeting differed among the segregation states of chromosomes. Furthermore, the NE reconstituted on segregated chromosomes exhibited active nucleocytoplasmic transport competency. These results indicate that the chromosome status changes after anaphase onset for recruiting NPC components.

## Introduction

The nuclear envelope (NE) spatially separates the chromatin from the cytoplasm in interphase eukaryotic cells to enable precise control. The NE consists of the outer nuclear membrane, the inner nuclear membrane (INM), and nuclear pore complexes (NPCs) that span both membranes to mediate nucleocytoplasmic transport. In vertebrates, the INM contains a distinct set of integral transmembrane proteins that interact with the nuclear lamina, representing a network of A-type and B-type lamins (for reviews, see Chow *et al.*, 2012; Holaska *et al.*, 2002; Pawar and Kutay, 2021).

During mitosis, the NE is disassembled but must reassemble on segregated chromosomes for proper nuclear function in the next interphase. During the prophase, the NE breaks down as follows: the NPC disassembles into subcomplexes of nucleoporins (Nups) (Kutay *et al.*, 2021), the lamina meshwork also disassembles (Gerace and Blobel, 1980; Mall *et al.*, 2012), and the membrane, which harbors membrane proteins, is absorbed into the mitotic endoplasmic reticulum (ER). After chromosome segregation, the NE starts to reassemble with membranes originating from the mitotic ER during telophase. This NE reassembly process must be coordinated with the assembly of NPCs, which are composed of approximately 30 Nups (Beck and Hurt, 2017; Hoelz *et al.*, 2016; Schwartz, 2016). This postmitotic NPC assembly occurs in an orderly, stepwise fashion (Kuiper *et al.*, 2023; Kutay and Hetzer, 2008; Otsuka and Ellenberg, 2018).

The early step of NE reassembly in human cells is characterized by specific pattern-like localization of INM proteins in two regions on telophase chromosomes that are termed the “core” region and the “noncore” region (Dechat *et al.*, 2004; Haraguchi *et al.*, 2000). A-type lamin and its binding INM proteins, such as emerin, localize to the core region, while B-type lamin and its binding INM proteins, such as lamin B receptor (LBR), localize to the noncore region. The exclusive accumulation of these proteins has also been shown in the early G1 interphase NE, which is correlated with NPC distribution; B-type lamin and LBR are localized to the NPC-enriched “pore-rich region”, and A-type lamin and emerin are localized to the NPC-depleted “pore-free region” (Maeshima *et al.*, 2010).

Barrier-to-autointegration factor (BAF) is a conserved DNA-binding protein that plays important roles in recruiting a number of INM proteins, including emerin, to the chromosome core region (Asencio *et al.*, 2012; Haraguchi *et al.*, 2001; Haraguchi *et al.*, 2008; Zhuang *et al.*, 2014). BAF is primarily phosphorylated during mitosis by vaccinia-related kinase 1 (VRK1) (Gorjánácz *et al.*, 2007). This phosphorylation reduces the abilities of BAF to bind to DNA and associate with LEM (LAP2–emerin–MAN1) domain proteins, including emerin (Bengtsson and Wilson, 2006; Margalit *et al.*, 2007; Molitor and Traktman, 2014; Nichols *et al.*, 2006). Phosphorylation of emerin during mitosis also reduces its associations with BAF and lamin (Emond-Fraser *et al.*, 2023; Hirano *et al.*, 2005). Dephosphorylation of BAF and LEM domain proteins, such as emerin, by PP2A-B55 and PP4 promotes complex formation and NE reassembly at the mitotic exit (Asencio *et al.*, 2012; Emond-Fraser *et al.*, 2023; Zhuang *et al.*, 2014). Moreover, Lem4/LEM-4L promotes the effective dephosphorylation of BAF by inhibiting VRK1 and by recruiting PP2 via its interaction (Asencio *et al.*, 2012; Gorjánácz, 2013). Therefore, dephosphorylation of BAF and INM proteins is a critical step in establishing the core region. LBR has a nucleoplasmic hydrophilic N-terminal region that interacts with various nuclear components, such as lamin B (Ye and Worman, 1994), DNA (Duband-Goulet and Courvalin, 2000; Ye and Worman, 1994), chromatin (Lu *et al.*, 2010; Takano *et al.*, 2002, 2004), histones (Hirano *et al.*, 2012; Polioudaki *et al.*, 2001), heterochromatin protein-1 (HP-1) (Ye and Worman, 1994; Ye *et al.*, 1997), and importin β (Lu *et al.*, 2010; Ma *et al.*, 2007). Phosphorylation of this LBR region by CDK1 and/or by SR protein kinases suppresses its association with these binding partners (Lu *et al.*, 2010; Takano *et al.*, 2002, 2004). Dephosphorylation of LBR may be required for chromosome targeting and accumulation in the noncore region. However, the mechanism involved in the accumulation of INM proteins and Nups in distinct chromosomal regions is unknown.

Many biochemical studies that used *Xenopus* egg extracts and sperm chromosomes have significantly contributed to our understanding of mitotic progression, including NE formation and NPC assembly. However, in this *in vitro* analysis system, the NE subdomain was not observed. Differential accumulation of INM proteins on chromosomes at the early step of NE assembly seems to be a general phenomenon in human cells (Maeshima *et al.*, 2006) and has been precisely described in HeLa cells (Clever *et al.*, 2012; Haraguchi *et al.*, 2001; LaJoie *et al.*, 2022). Therefore, we attempted to reconstitute an *in vitro* NE reassembly system in human cells that would recapitulate the phenomena observed in living mitotic cells. For this purpose, we used mitotically synchronized semi-intact cells treated with digitonin to permeabilize the plasma membrane while keeping the intracellular membranes intact.

Digitonin is widely used in immunostaining analysis to determine the topologies of transmembrane and nucleoplasmic proteins (Bodoor *et al.*, 1999; Söderqvist and Hallberg, 1994). Digitonin-permeabilized cells have been used for decades as a well-established cell-free reconstituted nucleocytoplasmic system (Adam *et al.*, 1990; Furuta *et al.*, 2014; Kose *et al.*, 2015), and they have been used in a variety of analyses to elucidate nucleocytoplasmic transport mechanisms (reviewed in Ding and Sepehrimanesh, 2021; Kassianidou *et al.*, 2019; Kose and Imamoto, 2014). These experimental systems are based on the use of the nonionic detergent digitonin, which is specific for cholesterol. Cholesterol is abundant in the plasma membrane but is less abundant in intracellular membrane systems (Lange *et al.*, 1989; van Meer *et al.*, 2008). Based on the membrane permeation selectivity of digitonin, a new biochemical method was established that separates the soluble nuclear fraction from the cytoplasmic fraction much more accurately than previously reported methods (Ogawa and Imamoto, 2021).

The development of an *in vitro* reconstitution assay system that recapitulates the phenomena observed in living cells would provide useful experimental tools for various biochemical analyses. One such example is an *in vitro* nucleocytoplasmic transport assay system that uses semi-intact cells, which was originally developed (Adam *et al.*, 1990). The use of this transport assay system allowed the identification of transport factors (Adam and Adam, 1994; Adam *et al.*, 1991; Chi *et al.*, 1995, 1996; Görlich *et al.*, 1994, 1995; Imamoto *et al.*, 1995a, 1995b; Kutay *et al.*, 1998; Melchior *et al.*, 1993; Moore and Blobel, 1994; Paschal and Gerace, 1995), various transport pathways (reviewed in Ding and Sepehrimanesh, 2021), and allowed the development of large-scale cargo identification system (Kimura *et al.*, 2017) or finding of novel stress-induced carrier protein (Kose *et al.*, 2012).

The use of mitotic semi-intact cells has the advantage of utilizing mitotic chromosomes, which are initial target sites for INM proteins and NE membranes that originate from the mitotic ER for postmitotic NE assembly. Herein, we reconstituted an *in vitro* NE assembly system that reflects previously reported phenomena observed in living cells. Studies using our *in vitro* system revealed the requirement for cytoplasmic factor(s) and energy sources, as well as different effects of mitotic progression states for the recruitment of INM proteins and NPC components to chromosomes.

## Materials and Methods

### Cell lines and culture

HeLa cells were maintained in Dulbecco’s modified Eagle’s medium (DMEM; Invitrogen, Waltham, MA, USA) supplemented with 10% fetal bovine serum (FBS; SH30070, Thermo Fisher Scientific, Waltham, MA, USA) at 37°C in a 5% CO_2_ atmosphere. HeLa cell line stably coexpressing LBR-Venus (LBR-YFP, yellow fluorescent protein) and super-enhanced cyan-emitting fluorescent protein, SECFP-emerin (CFP-emerin) was established as reported previously (Clever *et al.*, 2012; Sasaki *et al.*, 2008). For live imaging of living cells and time-lapse imaging of *in vitro* NE reconstitution reactions, cells were grown on glass-bottom dishes (10 mm diameter glass, P35G-0-10-C, MatTek, Ashland, MA, USA) one day before use, as described below. For cytosol preparation, HeLa-S3 cells were grown in suspension culture conditions in RPMI 1640 supplemented with 5% FBS as reported previously (Kose *et al.*, 2015) and as described in the section of NE reconstitution reaction.

### Live imaging of fluorescently labeled proteins expressed in intact HeLa cells

**Fig. 1:**
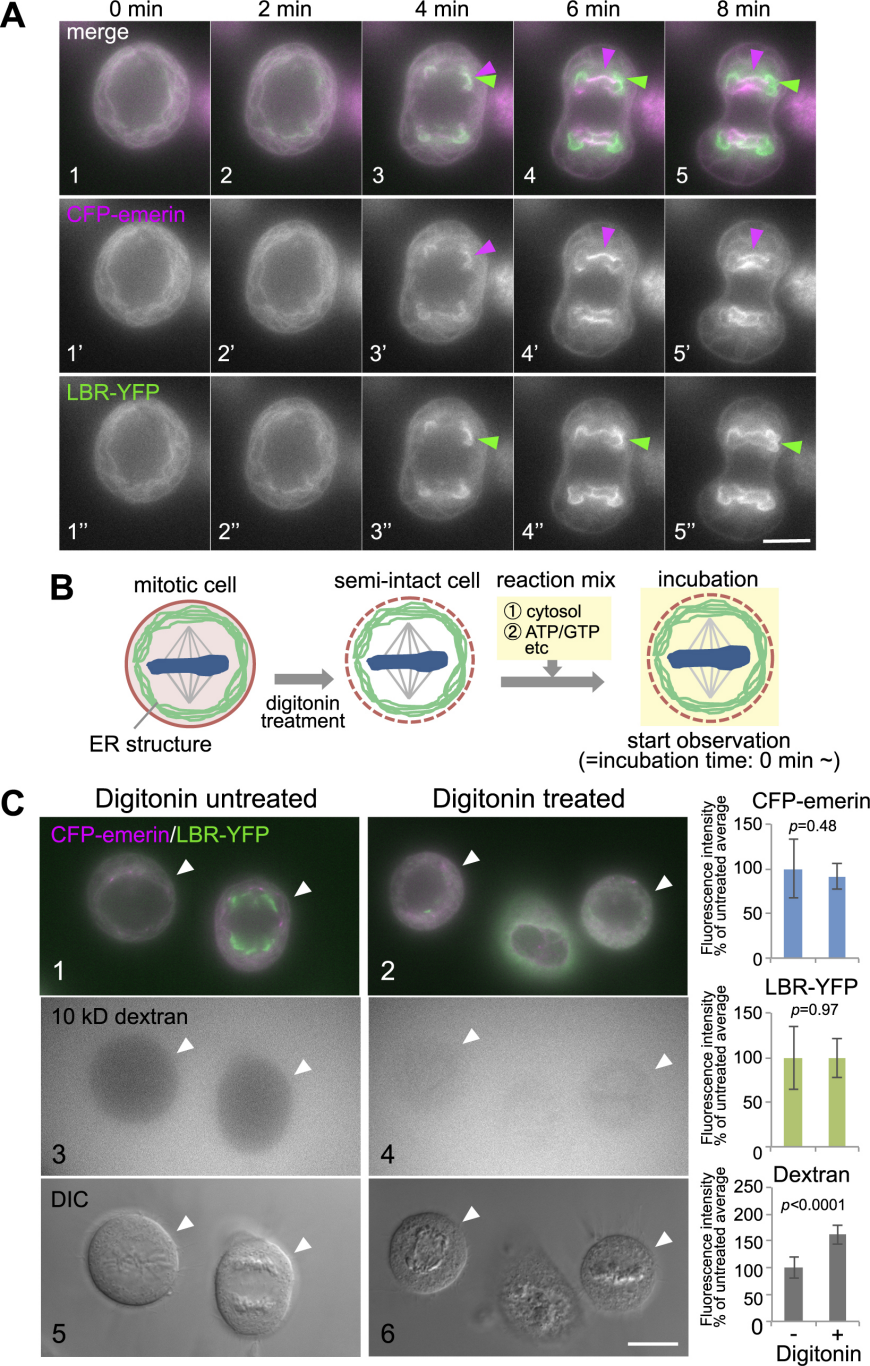
Subcellular localization of CFP-emerin and LBR-YFP in intact and semi-intact mitotic HeLa cells. (A) Live cell images of mitotic HeLa cells stably expressing CFP-emerin (magenta) and LBR-YFP (green) are shown at 2-min intervals. Arrowheads indicate chromosome recruitment sites (merge: 1–5; magenta: 1'–5', CFP-emerin; green: 1''–5'', LBR-YFP). Scale bar, 10 μm. (B) Schematic representation of the *in vitro* NE-reconstitution reaction. Mitotically synchronized HeLa cells coexpressing CFP-emerin and LBR-YFP (indicated as green lines) were treated with digitonin to permeabilize the plasma membrane (indicated as pink dashed lines). After washing out the cytosolic soluble materials, the permeabilized semi-intact cells were incubated with a reaction mixture composed of the cytosol, ATP regeneration system (ATP), GTP and other chemicals and proteins, and two INM proteins were monitored (see Materials and Methods). (C) Semi-intact cells produced by digitonin treatment contained membrane structures harboring fluorescently labeled INM proteins that were indistinguishable from those of untreated cells (magenta: CFP-emerin, green: LBR-YFP) (1, 2). The permeability of the plasma membrane was confirmed with 10-kDa dextran conjugated with Alexa Fluor^TM^ 594 (3, 4). Differential interference contrast (DIC) images of each field are presented (5, 6). Arrowheads indicate mitotic cells. Scale bar, 10 μm. The graphs show the fluorescence intensities of CFP-emerin, LBR-YFP and dextran conjugated with Alexa Fluor^TM^ 594 (Dextran) per unit area of metaphase cells as a percentage of the value for digitonin-untreated cells (mean ± SD). The cells were not treated with digitonin (n = 7) or treated with digitonin (n = 13).

The images were captured at 1-min intervals (shown at 2-min intervals in Fig. 1A) with a DeltaVision RT microscope (Applied Precision, Issaquan, WA, USA) using a PlanApo 60x/1.40 oil-immersion objective (Evident, Tokyo, Japan) and were acquired by softWoRx (Applied Precision). Images are presented without deconvolution. Images were exported in TIF format, linearly adjusted using softWoRx, cropped and resized with Photoshop (Adobe, San Jose, CA, USA) if needed.

### NE reconstitution reaction

For preparation of semi-intact cells, HeLa cells stably expressing fluorescent-labeled proteins were seeded at 1.5 × 10^5^ cells in a 35 mm glass-bottom dish with 10 mm diameter glass (P35G-0-10-C, MatTek) pretreated with poly-L-lysine. To enrich mitotic cells, cells were treated with 2 mM thymidine (T1895, Sigma-Aldrich, St. Louis, MO, USA) for 12–16 h, released by washing, incubated in DMEM supplemented with 10% FBS for 9–10 h, and subsequently subjected to digitonin treatment. Digitonin-permeabilized semi-intact cells were prepared as previously described (Adam *et al.*, 1990; Kose *et al.*, 2015) with minor modifications. In brief, cells were treated with 40 μg/mL digitonin (300410, Calbiochem, Merck, Darmstadt, Germany) in ice-cold transport buffer (TB) without EGTA [TB (-EGTA): 20 mM HEPES-KOH (pH 7.3), 110 mM potassium acetate, 2 mM magnesium acetate, 5 mM sodium acetate, 1 mM DTT and 1 μg/mL each of the aprotinin, leupeptin and pepstatin A] for 5 min on ice. After brief washing with ice-cold TB (-EGTA), the cells were incubated in the buffer for 10 min on ice. The permeability of the plasma membrane of the digitonin-treated cells was confirmed by the accessibility of 10-kDa dextran conjugated with Alexa Fluor^TM^ 594 (D22913, Invitrogen), as shown in Fig. 1C. Images were captured just after the addition of a reaction mixture and at 5-min intervals throughout the reactions at 30°C with a DeltaVision RT microscope (Applied Precision) using a PlanApo 60x/1.40 oil-immersion objective (Evident) and was acquired by softWoRx (Applied Precision). The reaction mixture was composed of (1) an ATP regeneration system (1 mM ATP, 5 mM creatine phosphate, and 20 U/mL creatine phosphokinase), (2) 0.1 mM GTP and (3) a cytosol (88% of the volume) in TB (-EGTA). The cyclin-dependent kinase (CDK) 1 inhibitor, RO-3306 (10 μM; 217699, Calbiochem) and Cy3 labeled bovine serum albumin tagged with SV40 T-antigen nuclear localization signal (Cy3-NLS-BSA) as a transport indicator, were further added where needed. For ATP/GTP absence conditions, apyrase was added at 0.1 U/mL. M-cyt was prepared as a cytosolic fraction from suspension cultures of HeLa-S3 cells as previously described (Kose *et al.*, 2015) with minor modifications. Briefly, HeLa-S3 cells were grown as a suspension culture in RPMI 1640 supplemented with 5% FBS, keeping the density at 2–5 × 10^5^ cells/mL. To prepare M-cyt, HeLa-S3 cells were mitotically arrested by treatment with 0.1 μg/mL nocodazole (Sigma) for 16–18 h. After washing with ice-cold phosphate-buffered saline, PBS(–) twice and then with ice-cold wash buffer [50 mM HEPES-KOH (pH 7.3), 110 mM potassium acetate, 2 mM magnesium acetate, 2 mM DTT] once, the cells were resuspended in an equal volume of ice-cold lysis buffer [5 mM HEPES-KOH (pH 7.3), 10 mM potassium acetate, 2 mM magnesium acetate, 2 mM DTT, 1 mM PMSF, 20 μM cytochalasin B, and 1 μg/mL each of aprotinin, leupeptin and pepstatin A] and left on ice for 10 min to swell the cells. The cells were lysed with a steel homogenizer (Dura-Grind, Wheaton, Millville, NJ, USA) by 10 strokes. After centrifugation at 16,000 × g for 15 min at 4°C, the supernatant was dialyzed against TB (-EGTA) without protease inhibitors at 4°C. The aggregates were removed by centrifugation at 16,000 × g for 15 min at 4°C. The protein concentration in the cytosolic fraction from mitotically synchronized HeLa-S3 cells was 15–17 mg/mL (3.0–3.5 mg/10^8^ cells), as determined by BCA Protein Assay Kits (23225, Thermo Fisher Scientific). After the reaction, the semi-intact cells were fixed with 3.7% formaldehyde in TB (-EGTA) for 10 min at room temperature (approximately 25°C) when immunofluorescence was needed.

### Indirect immunofluorescence

Immunofluorescence staining was performed as described previously (Funakoshi *et al.*, 2011) with minor modifications. Cells were treated with digitonin as described above and fixed in 3.7% formaldehyde in TB (-EGTA) for 10 min at RT either before or after the NE reconstitution reaction. Primary antibodies were diluted with 3% skim milk in PBS (–): mAb414 (MMS-120P, Covance, Berkeley, CA, USA), mouse anti-ELYS/Mel28 (embryonic large molecule derived from yolk sac; BMR00513, Bio Matrix Research, Kashiwa, Chiba, Japan), human anti-CREST (calcinosis, Raynaud’s phenomenon, esophageal dysmotility, sclerodactyly, and telangiectasia; 90C-CS1058, Fitzgerald, (Biosynth), Staad, Switzerland), and rabbit anti-H3S10P (06-570, Upstate, Lake Placid, NY, USA). The following secondary antibodies were purchased from Invitrogen: goat anti-mouse IgG Alexa Fluor^TM^ 594 conjugated, goat anti-human IgG Alexa Fluor^TM^ 647 conjugated, goat anti-rabbit IgG Alexa Fluor^TM^ 594 or Alexa Fluor^TM^ 647 conjugated. The DNA was counterstained with 4',6'-diamino-2-phenylindole (DAPI: 10236276001, Roche, Basel, Switzerland). The images were captured with a DeltaVision RT microscope (Applied Precision) as described in the live-imaging section.

### Quantification and statistical analysis

Fluorescence-based image analyses were performed using ImageJ software (National Institute of Health, Bethesda, MD, USA). A region of interest was selected. The mean intensity of each region was measured, and the background where there were no cells was subtracted. Statistical analyses were performed using Excel software (Microsoft, Redmond, WA, USA).

## Results and Discussion

### Reconstitution of postmitotic nuclear envelope formation in digitonin-permeabilized HeLa cells

To establish an *in vitro* system to analyze NE formation in human cells, we first isolated HeLa cells stably coexpressing two INM proteins, emerin and LBR, which were subsequently tagged with fluorescent proteins. Emerin was N-terminally tagged to SECFP (CFP-emerin), and LBR was C-terminally tagged to the yellow fluorescent protein derivative Venus (LBR-YFP). After NE breakdown, CFP-emerin and LBR-YFP were absorbed into the ER structures during mitosis (Fig. 1A1, 1', 1'' and Fig. S1A1–3). After anaphase onset, CFP-emerin and LBR-YFP simultaneously targeted the peripheral “noncore region” of segregated chromosomes (Fig. 1A3', 3''), followed by the accumulation of CFP-emerin in the “core region” (Fig. 1A4', 5'), while LBR-YFP further accumulated in the “noncore region” (see Fig. 1A4'', 5'' and Fig. S1A4–6). Although these phenomena have been previously described in detail (for reviews, see Clever *et al.*, 2013; Imamoto and Funakoshi, 2012; Kutay *et al.*, 2021).

To biochemically dissect postmitotic NE assembly in human cells, we attempted to reconstitute the postmitotic NE formation process using digitonin-permeabilized mitotic HeLa cells. We focused on NE subdomain formation, which is represented by the INM proteins emerin and LBR, as it is an initial step of NE formation in human cells. We generated mitotic semi-intact cells, as shown in Fig. 1B (see Materials and Methods). Dextran (10 kDa) rapidly penetrated the plasma membrane of digitonin-treated mitotic cells but not that of untreated cells, which confirmed successful treatment with digitonin (Fig. 1C). Notably, neither the localization nor the quantity of LBR-YFP or CFP-emerin in the metaphase of semi-intact cells changed upon digitonin permeabilization, as assessed by fluorescence intensity and localization (Fig. 1C).

NE formation occurs at telophase. We aimed to monitor the dynamics of the INM proteins in semi-intact cells to determine the properties of chromosomes that receive NE components for NE assembly. However, experimentally, we could not exactly monitor only telophase chromosomes without specific cell markers. Therefore, we permeabilized mitotic cells that was before and after anaphase onset, and compared them. We first examined the NE reassembly reaction in the presence of a cytosol that was prepared from asynchronous HeLa cells, which is generally used for nucleocytoplasmic transport assays (Adam *et al.*, 1990, 1991). However, the addition of this cytosol, even in the presence of ATP and GTP (ATP/GTP, Fig. S1C), did not support the recruitment of either INM protein in our *in vitro* assay (Fig. S1D–G). One possible reason was that this cytosol contained only limited amounts of factors required for NE assembly, such as BAF (Fig. S1B Lane 1), which may provide binding sites for emerin on chromosomes (Haraguchi *et al.*, 2001, 2008; Shimi *et al.*, 2004).

**Fig. 2:**
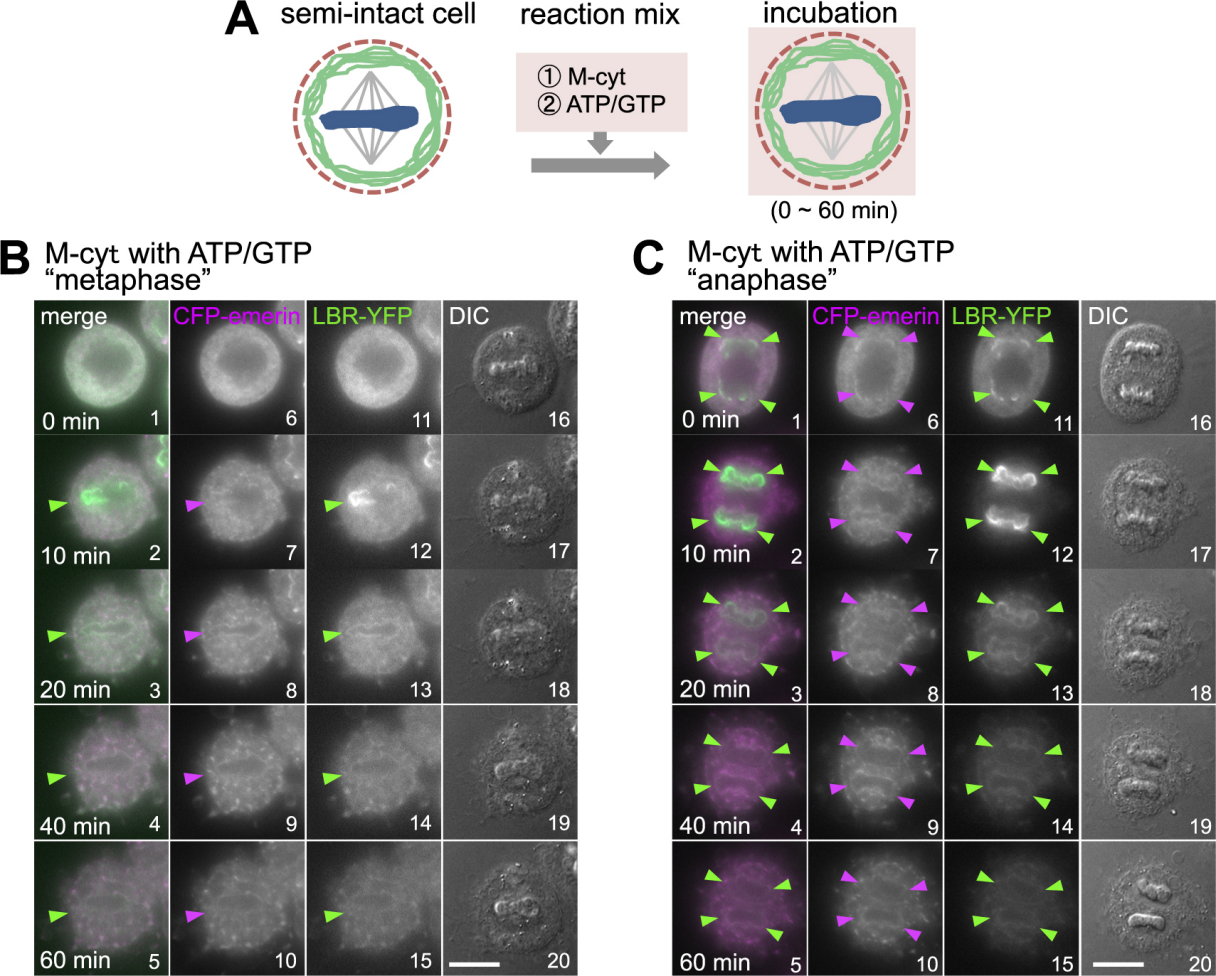
Transient accumulation of CFP-emerin and LBR-YFP on chromosomes in M-cyt. (A) Schematic representation of the *in vitro* assay of the NE reconstitution reaction with M-cyt and ATP/GTP. (B, C) Time-lapse images of the INM proteins in “metaphase” (B) or “anaphase” (C) semi-intact cell at the indicated incubation time points during the NE reconstitution reaction with M-cyt, ATP/GTP. Arrowheads indicate INM protein recruitment sites (magenta: CFP-emerin, green: LBR-YFP). Scale bars, 10 μm.

We therefore prepared a cytosol that was extracted from mitotically arrested HeLa S3 cells treated with nocodazole (M-cyt, see Methods) and performed an NE reconstitution assay in the presence of this cytosol (Fig. 2A). When a mixture of the M-cyt and ATP/GTP was added to semi-intact cells, LBR-YFP targeted chromosomes at different segregation stages of mitotic semi-intact cells; chromosomes were prepared from cells before anaphase onset (Fig. 2B, “metaphase”) and after anaphase onset (Fig. 2C, “anaphase”). However, the accumulation of LBR-YFP on chromosomes, irrespective of their segregation stage, was transient, and LBR-YFP was dispersed after 10 min of incubation (Fig. 2B, C: green arrowheads). CFP-emerin only weakly targeted chromosomes (Fig. 2B, C: magenta arrowheads).

**Fig. 3:**
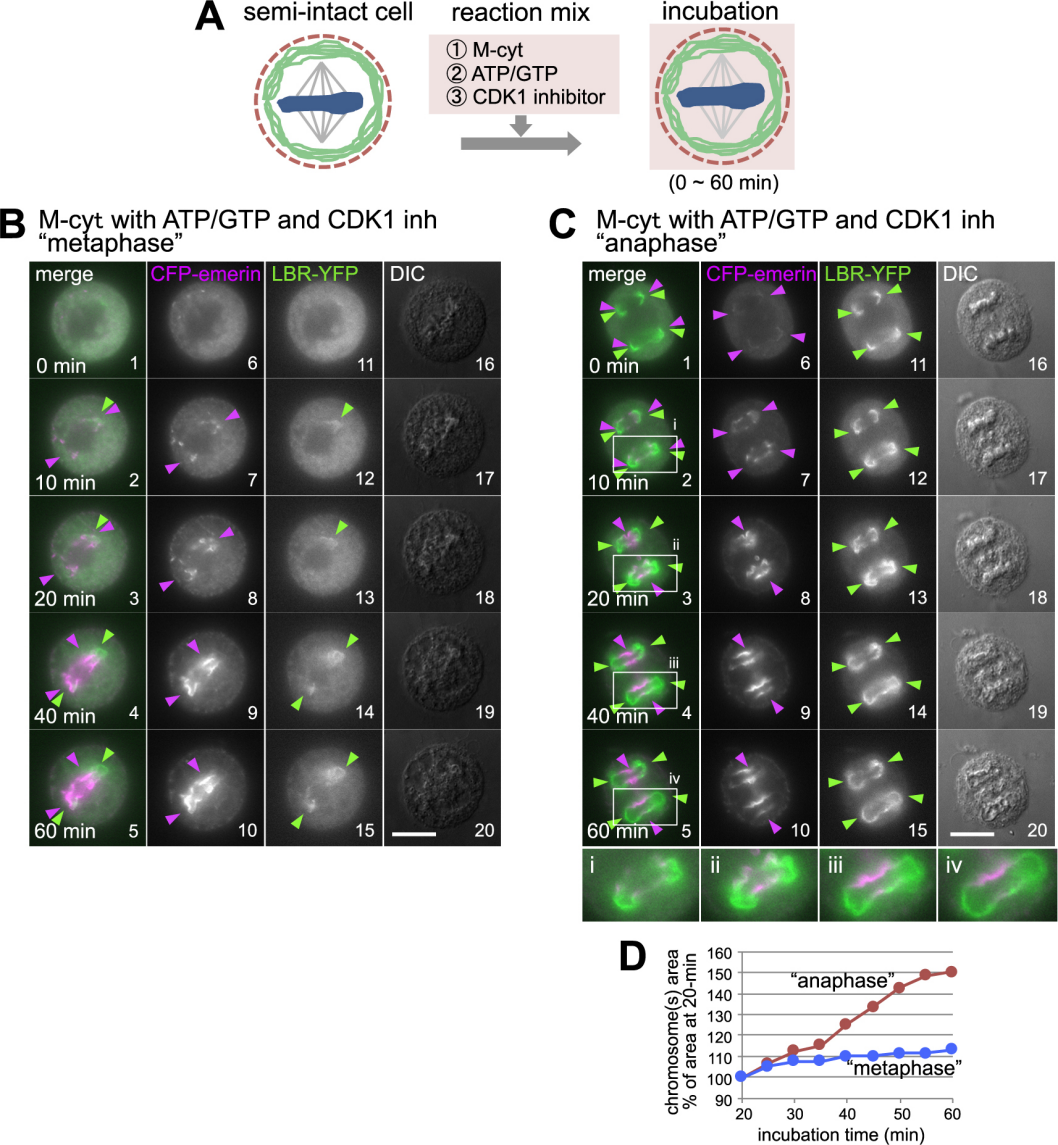
CDK inhibition enhances the chromosomal accumulation of CFP-emerin and LBR-YFP in M-cyt. (A) Schematic representation of the *in vitro* assay of the NE reconstitution reaction with M-cyt, ATP/GTP and a CDK1 inhibitor. (B, C) Time-lapse images of the INM proteins in “metaphase” (B) or “anaphase” (C) semi-intact cell at the indicated incubation time points during the NE reconstitution reaction with M-cyt, ATP/GTP and 10 μM RO-3306 (CDK1 inhibitor). Bottom panels (i–iv): 2 times magnification images of the boxed area in C. (D) The graph shows the change in total chromosomal area during the NE reconstitution reaction in B and C, expressed as a percentage of that at a 20-minute incubation point. The data is presented for “metaphase” (blue) and “anaphase” (red) chromosomes. Arrowheads indicate INM protein recruitment sites (magenta: CFP-emerin, green: LBR-YFP). Scale bars, 10 μm.

In living cells, once formed, the NE does not easily dissociate from chromosomes. We considered that some important elements could be lacking in our system. The M-cyt prepared from cells arrested by nocodazole should retain the high activity of cyclin B/Cdk. Cyclin-dependent kinase (CDK) 1 is the master regulator of mitosis that triggers mitosis by phosphorylation, and its activity is known to prevent NE reformation (Tseng and Chen, 2011; Vagnarelli and Earnshaw, 2012). We thus added the CDK1 inhibitor RO-3306 (Vassilev *et al.*, 2006) to reduce the kinase activity in the M-cyt and monitored the behavior of the INM proteins, as shown in Fig. 3A. We found that both INM proteins targeted mitotic chromosomes and were maintained on chromosomes. CFP-emerin and LBR-YFP both targeted the peripheral regions of “metaphase” chromosomes (Fig. 3B) and “anaphase” chromosomes (Fig. 3C). However, the two INM proteins accumulated differently; CFP-emerin accumulated in the central region, while LBR-YFP accumulated in the peripheral region. Such spatially separated accumulations mimic those in living telophase HeLa cells (Fig. 1A5', 5''; Haraguchi *et al.*, 2000).

The use of our present *in vitro* assay system apparently showed that NE components were recruited to chromosomes before chromosome segregation (“metaphase” chromosomes) in the presence of the M-cyt supplemented with RO-3306 and the energy sources. Mitotic processes may have proceeded artificially in our system when the CDK1 activity in the M-cyt was suppressed by the addition of RO-3306, and “metaphase” semi-intact cells may have progressed to a late anaphase/telophase-like phase, allowing the INM proteins to attach to chromosomes; in other words, the chromosome segregation process was decoupled in our *in vitro* assay. All the examined CDK inhibitors enhanced the accumulation of the INM proteins on chromosomes in the presence of the M-cyt (Fig. S2A, B), showing that the CDK activity in the M-cyt inhibited NE recruitment to chromosomes in our *in vitro* assay.

Next, we added λ protein phosphatase (PP), which has the opposite effect on CDKs, to the reaction mixture. As shown in Fig. S2C, the addition of λ PP to the reaction mixture of the M-cyt and ATP/GTP induced INM protein accumulation. The effect of λ PP was weakened after 20 min of incubation. Notably, the combined addition of λ PP and a CDK inhibitor with the M-cyt did not induce NE recruitment (Fig. S2D). In mitosis, the correct timing for switching to the sustained active phase of phosphatases and/or to the inactive phase of kinases, followed by maintaining a suitable balance, is important for the NE assembly process (for reviews, see Mochida and Hunt, 2012; Vagnarelli, 2021). A CDK inhibitor inhibits phosphorylation reactions, while a phosphatase mediates dephosphorylation reactions. Simultaneous addition of the CDK inhibitor and λ PP seemed to derange the activation or inactivation timing of kinase/phosphatase activity. For example, as shown in Fig. S3, histone H3 phosphorylation at Ser10 on metaphase chromosomes and its dephosphorylation on anaphase chromosomes were disturbed when a CDK inhibitor and λ PP were added simultaneously. The recruitment of ELYS to chromosomes was also disturbed.

When λ PP is added, it probably dephosphorylates CDK substrates, the majority of phosphoproteins in the prometaphase. In combination with phosphorylation suppression by a CDK inhibitor, λ PP may accelerate dephosphorylation at an incorrect time or even seemingly inhibit CDK activity. Mitotic events are driven by ordered dephosphorylation (McCloy *et al.*, 2015). The simultaneous addition of a CDK inhibitor and λ PP can disturb the precisely ordered dephosphorylation and coordinated events for NE reformation.

In our *in vitro* assay, emerin targeted and accumulated on chromosomes only when both M-cyt and CDK inhibitors were present, showing that cytosolic factor(s) contained in the M-cyt are required for chromosome targeting/accumulation (compare Fig. 3B, C and Fig. S4B). The reaction occurred on both “metaphase” and “anaphase” chromosomes. As mentioned in the Introduction, one of the most likely candidates for cytoplasmic factors required for emerin to bind to chromosomes is BAF, which is phosphorylated by VRK1 and released from chromatin-bound LEM domain proteins to the cytoplasm (Gorjánácz *et al.*, 2007; Jamin *et al.*, 2014; Molitor and Traktman, 2014). In the M-cyt, BAF is phosphorylated but needs to be dephosphorylated to recruit emerin to chromosomes, thus requiring CDK inhibition.

In the case of LBR, its targeting/accumulation to “metaphase” chromosomes required the M-cyt, while its targeting/accumulation to “anaphase” chromosomes did not (Fig. 3B, C and Fig. S4B). The cytosol-independent accumulation of LBR-YFP on “anaphase” chromosomes required ATP/GTP (Fig. S4C). This accumulation was hindered by PP inhibitors (Fig. S4Dd, e) but not by kinase inhibitors (Fig. S4Db, c). The accumulation of LBR-YFP was suppressed by inhibitor-2 (Fig. S4De), a PP1-specific inhibitor, suggesting that residual PP (probably PP1) in semi-intact cells contributed to LBR-YFP accumulation on “anaphase” chromosomes.

Previous studies have suggested that protein phosphatase 1 (PP1) plays important roles at the mitotic exit (Huguet *et al.*, 2022; Ito *et al.*, 2007; Vagnarelli *et al.*, 2011). Repo-Man, a specific regulatory subunit of PP1γ, plays crucial roles. At anaphase onset, Repo-Man/PP1γ becomes enriched on the segregating chromatin and promotes histone H3 dephosphorylation at Thr3, Ser10 and Ser28. Dephosphorylation of histone H3 at Ser10 seems to be involved in the regulation of H3 binding to HP1, which is known as an LBR binding target on chromosomes. A portion of Repo-Man/PP1γ targets the periphery of chromosomes slightly later in the anaphase, and such peripheral Repo-Man can recruit the nucleocytoplasmic machinery, such as importin β and Nup153 (Vagnarelli *et al.*, 2011; Vagnarelli and Earnshaw, 2012). Our observation of the cytosol-independent targeting of LBR-YFP to “anaphase” chromosomes, which is likely to be promoted by residual PP1, was consistent with these reports. LBR required cytosolic factors to target/accumulate on “metaphase” chromosomes, probably because Repo-Man/PP1γ is not sufficiently enriched without cytosolic factors. In addition, it is possible that PPs contained in the M-cyt start to dephosphorylate kinase substrates when cyclin B/CDK1 activity is reduced.

Our observations regarding the cytosolic requirements of CFP-emerin and LBR-YFP to target/accumulate on chromosomes in the *in vitro* NE reconstitution assay were consistent with previously reported findings in living cells. However, the molecular basis of the ATP/GTP requirement remains unknown.

### Nuclear transport competency of the reconstituted NEs

**Fig. 4:**
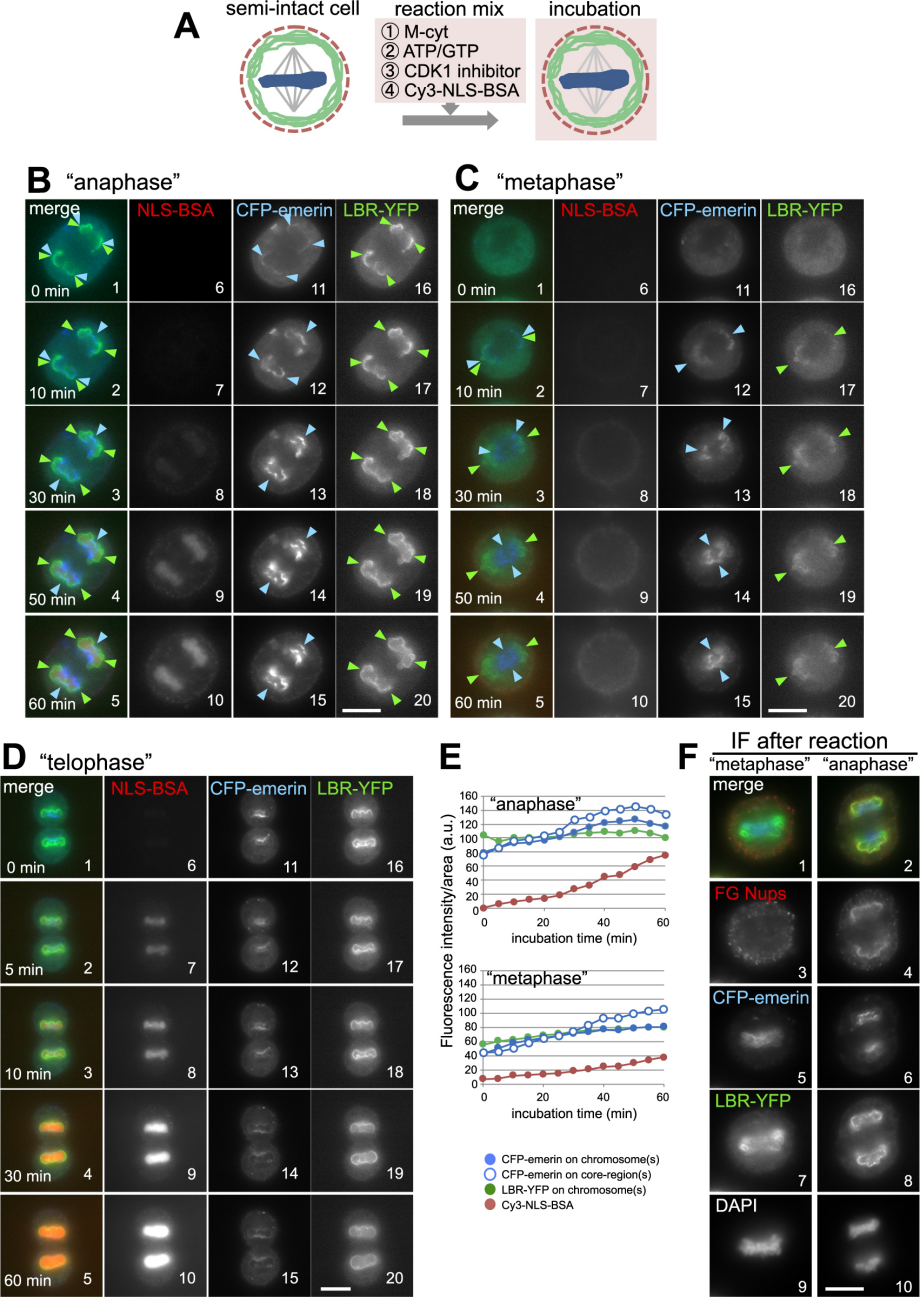
Nuclear transport activity of the reconstituted NE. (A) Schematic representation of the *in vitro* nuclear transport assay of reconstituted NE with M-cyt, ATP/GTP, and a CDK1 inhibitor. Cy3-NLS-BSA was added as a nucleocytoplasmic transport indicator. (B–D) Time-lapse images of the INM proteins and Cy3-NLS-BSA (NLS-BSA) at the indicated incubation time points during the NE reconstitution reaction as described in A: in “anaphase” (B), “metaphase” (C) and “telophase” semi-intact cells (D; a positive control for nuclear transport). Arrowheads indicate INM protein recruitment sites (blue: CFP-emerin, green: LBR-YFP). (E) Fluorescence intensity per unit area of CFP-emerin (blue circles), LBR-YFP (green circles) and Cy3-NLS-BSA (red circles) for entire chromosome(s) during the NE reconstitution reaction in B (“anaphase”) and C (“metaphase”). The blue open circles show the fluorescence intensity per unit area of CFP-emerin at the core region. (F) Images of FG Nups (3, 4), CFP-emerin (5, 6) and LBR-YFP (7, 8) after NE reconstitution (40 min) as in A without Cy3-NLS-BSA. FG Nups were visualized by immunofluorescence with mAb414. The DNA was counterstained with DAPI (9, 10). The upper panels show merged images of FG Nups (red), CFP-emerin (blue), and LBR-YFP (green) (1, 2). Scale bars, 10 μm.

We noticed that “anaphase” chromosomes swelled after the accumulation of CFP-emerin in their central regions (Fig. 3Ci–iv, D). This finding suggested that active nuclear transport might have taken place when the NE was reconstituted in our *in vitro* reaction. To test this possibility, we examined the nuclear transport of reconstituted NEs by monitoring the nuclear accumulation of Cy3-labeled SV40 large T-antigen NLS-conjugated BSA (Cy3-NLS-BSA) during the NE reconstitution reaction, as shown in Fig. 4A. NLS-BSA is known to be transported into the nucleus via the importin α/importin β-dependent pathway, and fluorescently labeled NLS-BSA is used as an indicator of a functional NE (Haraguchi *et al.*, 2000; Hetzer *et al.*, 2000). Cy3-NLS-BSA immediately accumulated (within 5 min) in the assembled nucleus of digitonin-permeabilized cells under our conditions (as shown in the control “telophase”, Fig. 4D7, Movie S1). When “anaphase” chromosomes were monitored, Cy3-NLS-BSA began to accumulate in the reconstituted nuclei after CFP-emerin accumulated in the central region (see Fig. 4B8 for the time point after 30 min; Fig. 4E and Movie S2). The appearance of detectable transport activity following the accumulation of emerin in the core region of chromosomes is consistent with observations in living cells (Haraguchi *et al.*, 2000). These results indicate that the reconstituted nuclei in our system gained nuclear transport activity. This transport activity of soluble proteins also suggested that functional NPCs were assembled in our system and that the reconstituted NE membranes were intact. On the other hand, no or very low transport activity was observed by monitoring “metaphase” chromosomes, even when LBR-YFP and CFP-emerin were targeted (Fig. 4C, E; Movie S3). Further examination by electron microscopy is required to confirm the complete enclosure of the reconstructed NE membrane because a previous report suggested that nuclear transport activity could be detected prior to the completion of the NE enclosure in the core region (Haraguchi *et al.*, 2008).

The above results suggested that mature NPCs were assembled on reconstituted NEs on “anaphase” chromosomes but not on “metaphase” chromosomes, even though both of chromosomes were incubated with the same cytosol. We examined whether NPCs were assembled on chromosomes after the NE reconstitution reaction in the presence of the M-cyt supplemented with ATP/GTP and a CDK1 inhibitor by immunofluorescence using an mAb414 antibody, which reacts with FG Nups, the main components of the transport channel of the NPC. As shown in Fig. 4F, “anaphase” chromosomes acquired mAb414 (FG Nups)-positive structures, whereas “metaphase” chromosomes were negative for the mAb414 signal (Fig. 4F3, 4). These results suggested that NPCs indeed assembled into the NE on “anaphase” chromosomes but not on “metaphase” chromosomes, explaining why only “anaphase” chromosomes were nuclear transport competent.

### Different behaviors of the scaffold NPC components on “metaphase” and “anaphase” chromosomes

The above results raise an intriguing possibility that a chromosome state switches after anaphase onset to recruit the NPC components Nups. Disassembled Nups and nuclear membrane proteins should be spatiotemporally coordinated to form functional nuclei at the mitotic exit. In the early step of NE reassembly, recruitment of ELYS/Mel28, a DNA-binding protein, to segregated chromosomes is known to trigger mitotic NPC assembly (Galy *et al.*, 2006; Gillespie *et al.*, 2007; Rasala *et al.*, 2006). ELYS/Mel28 binds to the Nup107–160 subcomplex and subsequently recruits this subcomplex to chromosomes (Doucet *et al.*, 2010; Franz *et al.*, 2007). The Nup107–160 subcomplex then recruits the transmembrane component Pom121 and the other scaffold Nup93 complex (Dultz *et al.*, 2008), which is subsequently integrated with peripheral Nups to form new NPCs (Dultz *et al.*, 2008; Rasala *et al.*, 2008; Walther *et al.*, 2003). ELYS/Mel28 is also important for the effective recruitment of INM proteins to chromosomes in HeLa cells during mitosis (Clever *et al.*, 2012) and to the NE during the interphase (Mimura *et al.*, 2016).

**Fig. 5:**
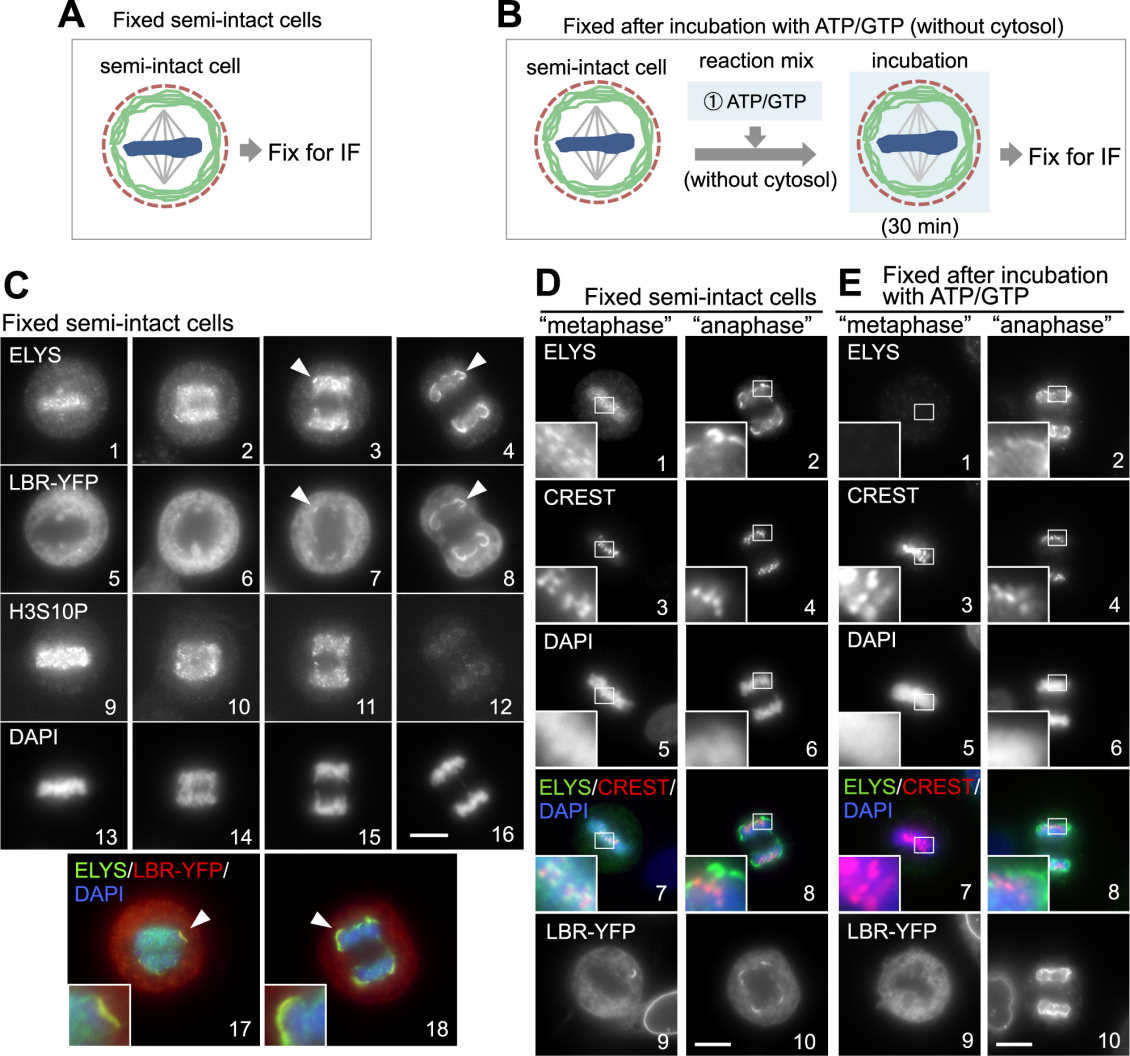
Dynamics of LBR and ELYS/Mel28 on mitotic chromosomes. Schematic representation of immunofluorescence for semi-intact cells: (A) fixed immediately after digitonin permeabilization or (B) fixed after incubation in buffer containing ATP/GTP in the absence of the cytosol. (C) Images of ELYS/Mel28 (1–4), LBR-YFP (5–8), and H3S10P (9–12) at different mitotic stages in fixed semi-intact cells as in A. ELYS/Mel28 and H3S10P were visualized by immunofluorescence. Arrowheads indicate colocalization of LBR-YFP and ELYS/Mel28 on chromosomes. The DNA was counterstained with DAPI (13–16). Merged images of ELYS/Mel28 (green), LBR-YFP (red) and DNA (DAPI, blue) (17, 18). (D, E) Images of LBR-YFP (9, 10), ELYS/Mel28 (1, 2) and CREST (3, 4) in semi-intact cells fixed immediately after permeabilization, as described in A (D), or after incubation in buffer with ATP/GTP in the absence of the cytosol, as described in B (E). The DNA was counterstained with DAPI (5, 6). The window in the panel shows 3.75 times magnified images. Scale bars, 10 μm.

We examined the localization of endogenous ELYS/Mel28 on chromosomes at different mitotic segregation stages by immunofluorescence immediately after digitonin permeabilization, as shown in Fig. 5A. Most of the endogenous ELYS/Mel28 in semi-intact cells was detected as dots on “metaphase” chromosomes (Fig. 5C1, D1), reflecting kinetochore localization, as previously reported for intact cells (Franz *et al.*, 2007; Rasala *et al.*, 2006). Indeed, fluorescent signal of ELYS/Mel28 overlap with CREST, a centromere marker (Fig. 5D3, D7). ELYS/Mel28, which localized with the kinetochore, became more dispersed as the chromosome segregation proceeded, and eventually, it colocalized with LBR-YFP in the peripheral noncore region on “anaphase” chromosomes (Fig. 5C3, 4, 17, 18, arrowheads). To determine the dynamics of ELYS/Mel28 on chromosomes, semi-intact cells were incubated in a buffer (minus cytosol) supplemented with ATP/GTP prior to immunostaining (Fig. 5B). ELYS/Mel28 was released from “metaphase” chromosomes during incubation (compare Fig. 5D1 and E1), but it was not released from “anaphase” chromosomes under the same conditions (Fig. 5E2), indicating that the association state of ELYS/Mel28 with chromosomes becomes more stable after anaphase onset. We also observed the behavior of Nup107 and found that it was also released from “metaphase” chromosomes but not from “anaphase” chromosomes, as was the case for ELYS/Mel28, under the same conditions (Fig. S5A, B). The stable associations of ELYS/Mel28 and Nup107 with “anaphase” chromosomes might affect the assembly and transport competency of the NPC (see above).

Several Nup107–160 complex components, such as ELYS/Mel28, relocate to kinetochores after NE breakdown (Belgareh *et al.*, 2001). FRAP experiments on Nup133 revealed that its interaction with the kinetochore was not stable (Belgareh *et al.*, 2001). *In vitro* experiments using *Xenopus* materials showed that ELYS/Mel28 was required for mitotic spindle formation, in addition to its function in NPC assembly (Yokoyama *et al.*, 2014). The release of ELYS/Mel28 from the kinetochore was suppressed by kinase inhibitor staurosporine (Fig. S5C), suggesting that kinase activity at the kinetochore is maintained during the metaphase. Kinetochores are mechanosensors that control the stability of microtubule attachment to favor the biorientation of sister chromatids (reviewed in Foley and Kapoor, 2013). Kinases that are localized on metaphase chromosomes, such as Aurora B kinase, Plk1 (polo-like kinase 1), Mps1 (monopolar spindle 1), Bub1 (budding uninhibited by benzimidazoles 1), Haspin and CDK1 (Funabiki and Wynne, 2013; Manic *et al.*, 2017), could be candidates that are responsible for these phenomena, although further studies are needed to confirm this possibility.

We previously reported phosphorylation-dependent interactions between ELYS/Mel28 and LBR (Clever *et al.*, 2012; Mimura *et al.*, 2016). LBR harbors phosphorylation sites in its N-terminal region, and its phosphorylations promote interactions with binding partners (Clever *et al.*, 2012; Lu *et al.*, 2010; Mimura *et al.*, 2016; Takano *et al.*, 2002, 2004). LBR phosphorylation at residues Ser71 and Ser86, which are CDK target sites (Lu *et al.*, 2010; Nikolakaki *et al.*, 1997; Tseng and Chen, 2011), and at serine residues within the RS domain, which are serine/arginine protein kinase target sites (Nikolakaki *et al.*, 1997), promoted interactions with ELYS/Mel28 (Mimura *et al.*, 2016). ELYS/Mel28 may keep the phosphorylated LBR away from chromosomes before segregation and recruit it to chromosomes when the phosphorylation state is reduced after anaphase onset.

NPC assembly proceeds via stepwise interactions among subcomplexes. In metazoans, two distinct NPC assembly pathways have been suggested, interphase and mitotic NPC assembly pathways. The former is *de novo* pathway in which NPCs are formed into a sealed nuclear membrane bilayer, while the latter is relatively fast reassembly pathway in which NPCs are formed on mitotic chromosomes with dispersed subcomplexes. The ER network first contacts chromatin as sheets or tubules that flatten and expand to directly form a NE sheet with NPCs assembling on the chromatin (Anderson and Hetzer, 2007; LaJoie and Ullman, 2017; Wandke and Kutay, 2013). A recent study has indicated that NPCs start to assemble precursors, which are detected as electron-dense material in small membrane holes of the fenestrated reforming NE, after which they extend to larger NPC-sized channels (Otsuka *et al.*, 2018; Bilir *et al.*, 2019). Otsuka and colleagues demonstrated that the initial phases of mitotic NPC assembly differ from those of interphase assembly, which initially occurs on the nucleoplasmic side of the NE (Otsuka *et al.*, 2016, 2023). In mitotic NPC assembly, Nups of Y-complexes have been reported to be associated with the mitotic ER (Chou *et al.*, 2021; Gandhimathi *et al.*, 2023). Y-complexes on the mitotic ER might act as seeds for NPC assembly on the NE formed around chromosomes.

As reported above, the regulation of the ER morphology and its interactions with Nups and chromosomes also important for the formation of functional nuclear membranes and interphase nuclei. It is plausible that some elements or factors that are acquired by chromosomes after anaphase onset are responsible for functional NPC formation.

### Perspectives

In our *in vitro* assay, the targeting/accumulation of LBR on “metaphase” chromosomes required energy sources and an M-cyt with suppressed CDK activity (Fig. 3B), while the requirements for cytoplasmic factors for LBR accumulation on “anaphase” chromosomes were ambiguous (Fig. S4B). Regardless of the degree of cytosolic dependency, LBR accumulation always requires ATP/GTP. GTP hydrolysis may be needed to release LBR from importin β as a function of RanGEF RCC1, which is associated with chromosomes. We confirmed that excess importin β inhibited the cytosol-independent recruitment of LBR to “anaphase” chromosomes in semi-intact cells (Fig. S4E), as was also shown in previous reports using an *in vitro* *Xenopus* system (Harel *et al.*, 2003; Lau *et al.*, 2009).

Semi-intact cells are an excellent system for analyzing factors for nucleocytoplasmic transport because their nuclear membrane structures and functions are compromised (Adam *et al.*, 1990). On the other hand, our present system uses semi-intact mitotic cells, in which nuclear membranes and nuclear structures are absent. Therefore, researchers may claim that it is difficult to perform analyses using this *in vitro* system. But as discussed above, phenomena observed in our *in vitro* system, such as NE subdomain formation exemplified by the distribution of two INM proteins, and the nuclear transport ability of the reassembled NE recapitulated phenomena observed in living cells. In addition, the requirement for cytoplasmic factors and kinase/phosphatase activities for chromosome targeting of INM proteins can be explained by previously reported results that was based on the observations in living cells. Therefore, we think the phenomena observed in our system could reflect those observed in living cells. Development of present NE reconstitution system would expand the possibilities for using semi-intact cells. However, because the mitotic phase progresses rapidly and phases change within a short period of time, more detailed phase classification (metaphase, anaphase, telophase, etc.) may be necessary to identify responsible molecules.

## Data Availability

The supporting information for this article is available in J-STAGE Data.

## Abbreviations

BAF: barrier-to-autointegration factor
BSA: bovine serum albumin
Bub1: budding uninhibited by benzimidazoles 1
CDK: cyclin-dependent kinase
CREST: calcinosis, Raynaud’s phenomenon, esophageal dysmotility, sclerodactyly, and telangiectasia
DAPI: 4',6'-diamidino-2-phenylindole
DIC: differential interference contrast
DMEM: Dulbecco’s modified Eagle’s medium
ELYS: embryonic large molecule derived from yolk sac
ER: endoplasmic reticulum
FBS: fetal bovine serum
HP-1: heterochromatin protein-1
INM: inner nuclear membrane
LBR: lamin B receptor
LEM: LAP2–emerin–MAN1
Mps1: monopolar spindle 1
NE: nuclear envelope
NLS: nuclear localization signal
NPC: nuclear pore complex
Nup: nucleoporin
PBS: phosphate-buffered saline
Plk1: polo-like kinase 1
PP: protein phosphatase
SECFP: super-enhanced cyan-emitting fluorescent protein
TB: transport buffer
VRK1: vaccinia-related kinase 1
YFP: yellow fluorescent protein
