# Supplementary material for: Reconstitution of nuclear envelope subdomain formation on mitotic chromosomes in semi-intact cells
Source: Cell Struct Funct. 2024 Jun 4;49(2):31–46. doi: 10.1247/csf.24003 (PMC11926407; doi:10.1247/csf.24003)
Supplement: Supplementary file 4 — Supplementary Materials [file csf_49_24003_4.zip › 49_24003_4.docx]

**Supplementary materials**

**Supplementary Materials and Methods**

*Cell line*

HeLa cells stably expressing Venus-Nup107 were established as reported previously (Maeshima *et al*., 2010).

*Asynchronous cell cytosol preparation*

Asynchronous cytosol was prepared as previously described (Kose, *et al*., 2015) and in the section of NE reconstitution reaction without synchronization with nocodazole.

*Live imaging of fluorescently labeled proteins expressed in intact HeLa cells*

HeLa cells stably coexpressing LBR-YFP and CFP-emerin were grown on glass-bottom dishes and labeled with ER-Tracker^TM^ Blue-White DPX (E12353, Invitrogen). For Fig. S1A, images were captured with a DeltaVision RT microscope (Applied Precision) using a PlanApo 60x/1.40 oil-immersion objective (Olympus) and acquired by softWoRx (Applied Precision). The images were subjected to deconvolution via softWoRx (Applied Precision).

*Inhibitors and recombinant proteins used for the NE reconstitution reaction*

The following inhibitors or peptides were used: staurosporine (569397, Calbiochem), okadaic acid (154-01651, Wako), human recombinant protein phosphatase (PP) 1 inhibitor-II (539638, Calbiochem), alsterpaullone (A4748, Sigma), λ PP (P0753S, NEB), and bacterially expressed and purified recombinant Importin β (Tahara *et al.*, 2008). The concentrations used are described in the figure legends.

*Western blotting*

Cytosol fractions prepared from HeLa-S3 cells were subjected to SDS-PAGE. The proteins that were transferred to PVDF membranes were detected with the following primary antibodies diluted with 3% skim milk in Tris buffered saline with Tween 20: rabbit anti-BAF (sc33787), mouse anti-cyclin B1 (sc-245), mouse anti-lamin A/C (sc-7292), (Santa Cruz Biotechnology), mouse anti-NuMA (D215-3, MBL, Japan), rabbit anti-Nup107 (A301-787A, Bethyl), and mouse anti-Ran (610341, BD Transduction).

*Movie*

All the movies were constructed by softWoRx (Applied Precision) with 13 still images (during 60 min of the NE reconstitution reaction at 5 min intervals). A movie is shown at 4 frames per second.

**Supplementary Figure Legends**

Fig. S1 Distributions of CFP-emerin and LBR-YFP in live cells and semi-intact cells during *in vitro* reaction with asynchronous cytosol and ATP/GTP. (A) Live cell images of mitotic HeLa cells: merged images of LBR-YFP (green) and ER-tracker (blue) (1, 4), ER-tracker (2, 5), and merged images of LBR-YFP (green) and CFP-emerin (red) (3, 6). The images were subjected to deconvolution. Scale bars, 10 μm. (B) Western blot of cytosolic proteins in asynchronous (Asynch-cyt, Lane 1) or mitotic (M-cyt, Lane 2) HeLa cells with antibodies against lamin A/C, NuMA, Nup107, BAF, cyclin B1, and Ran. (C) Schematic representation of the *in vitro* NE reconstitution reaction with asynchronous cytosol and ATP/GTP. (D-G) Time-lapse images of CFP-emerin and LBR-YFP expressed in semi-intact cells at the indicated incubation time points during the NE reconstitution reaction with asynchronous cytosol supplemented with ATP/GTP (D, E) or without ATP/GTP (F, G). DIC images of each field are presented. Arrowheads indicate INM protein recruitment sites (magenta: CFP-emerin, green: LBR-YFP). Scale bars, 10 μm.

Fig. S2 Accumulations of INM proteins on chromosomes during *in vitro* reaction with M-cyt and ATP/GTP were induced by CDK inhibitor or 𝜆 PP, but not by simultaneously addition of them. Time-lapse images of LBR-YFP and CFP-emerin at the indicated incubation time points during the NE reconstitution reaction with M-cyt and ATP/GTP supplemented with the indicated kinase inhibitors (A: 5 μM alsterpaullone, B: 10 μM roscovitine), 𝜆 PP (C: 2 U/μL) or both (D: 5 μM alsterpaullone and 2 U/μL 𝜆 PP). Arrowheads indicate INM protein recruitment sites (magenta: CFP-emerin, green: LBR-YFP). Scale bars, 10 μm.

Fig. S3 Distributions of ELYS/Mel28 and H3S10P in semi-intact cells after *in vitro* reaction with M-cyt supplied with ATP/GTP and CDK inhibitor and 𝜆 PP. ELYS/Mel28 and H3S10P were visualized by immunofluorescence in semi-intact cells stably expressing CFP-emerin and LBR-YFP fixed immediately after digitonin permeabilization (A) or after reaction for 60 min in M-cyt supplemented with ATP/GTP and both of CDK inhibitor and 𝜆 PP (B: 5 μM alsterpaullone, 2 U/μL) or CDK inhibitor (C: 5 μM alsterpaullone) or 𝜆 PP (D: 2 U/μL). Scale bars, 10 μm.

Fig. S4 Cytosol-independent accumulation of LBR-YFP on “anaphase” chromosomes in semi-intact cells. (A) Schematic representation of time-lapse imaging at indicated incubation time in buffer with or without ATP/GTP in the absence of the cytosol (i), and further supplied inhibitors or Importin β (ii). (B, C) Time-lapse images of CFP-emerin and LBR-YFP in semi-intact cells incubated for 30 min in buffer supplemented with ATP/GTP (B) or without ATP/GTP (C) in the absence of the cytosol as described in A-(i). Arrowheads indicate INM protein recruitment sites (magenta: CFP-emerin, green: LBR-YFP). (D) PP inhibitors prevented the cytosol-independent recruitment of LBR-YFP to “anaphase” chromosomes. Images of LBR-YFP in semi-intact cells incubated for 30 min in buffer supplemented with ATP/GTP (a) and further supplied kinase inhibitors (b: Stsp, 10 μM staurosporine; c: RO, 10 μM RO-3306) or phosphatase inhibitors (d: OA, 5 μM okadaic acid, e: I-2, 2 μM PP1 inhibitor-2) as in A-(ii). (E) An excess amount of Importin β inhibited the cytosol-independent accumulation of LBR-YFP to “anaphase” chromosomes. Images of LBR-YFP in semi-intact cells fixed immediately after permeabilization (a: semi-intact cell), or after incubation for 30 min in buffer with ATP/GTP (b: (-)), and further 80 μM Importin β (c: Imp-β) as shown in A-(ii). The DNA was counterstained with DAPI. Scale bars, 10 μm.

Fig. S5 Dynamics of Nup107 and ELYS/Mel28 on mitotic chromosomes. (A, B) Images of Venus-Nup107 (YFP-Nup107: 3, 4) in semi-intact cells stably expressing Venus-Nup107 fixed immediately after digitonin permeabilization (A) or fixed after incubation in buffer supplemented with ATP/GTP in the absence of the cytosol (B). ELYS/Mel28 was visualized by immunofluorescence (5, 6). The DNA was counterstained with DAPI (7, 8). (C) Kinase inhibitor suppressed the ELYS/Mel28 releasing from “metaphase” chromosomes. Semi-intact cells were fixed immediately after digitonin permeabilization (a) or after reaction for 30 min in buffer supplemented with ATP/GTP in the absence of the cytosol as in B (b) or further added staurosporine at 10 μM (c). ELYS/Mel28 (4-6) and H3S10P (7-8) were visualized by immunofluorescence. The DNA was counterstained with DAPI (10-12). Scale bars, 10 μm.

Movie S1-3. Transport competency of the reconstituted NE. Dynamics of LBR-YFP (green), CFP-emerin (blue) and Cy3-NLS-BSA (red) during the NE reconstitution reaction in semi-intact cells at telophase (1), anaphase (2), or metaphase (3). Still images of movies S1, 2, 3 were used for Figs. 4 D, B. C, respectively. Scale bars, 10 μm.
