# Supplementary material for: Reconstitution of nuclear envelope subdomain formation on mitotic chromosomes in semi-intact cells
Source: Cell Struct Funct. 2024 Jun 4;49(2):31–46. doi: 10.1247/csf.24003 (PMC11926407; doi:10.1247/csf.24003)

Supplementary figure 1

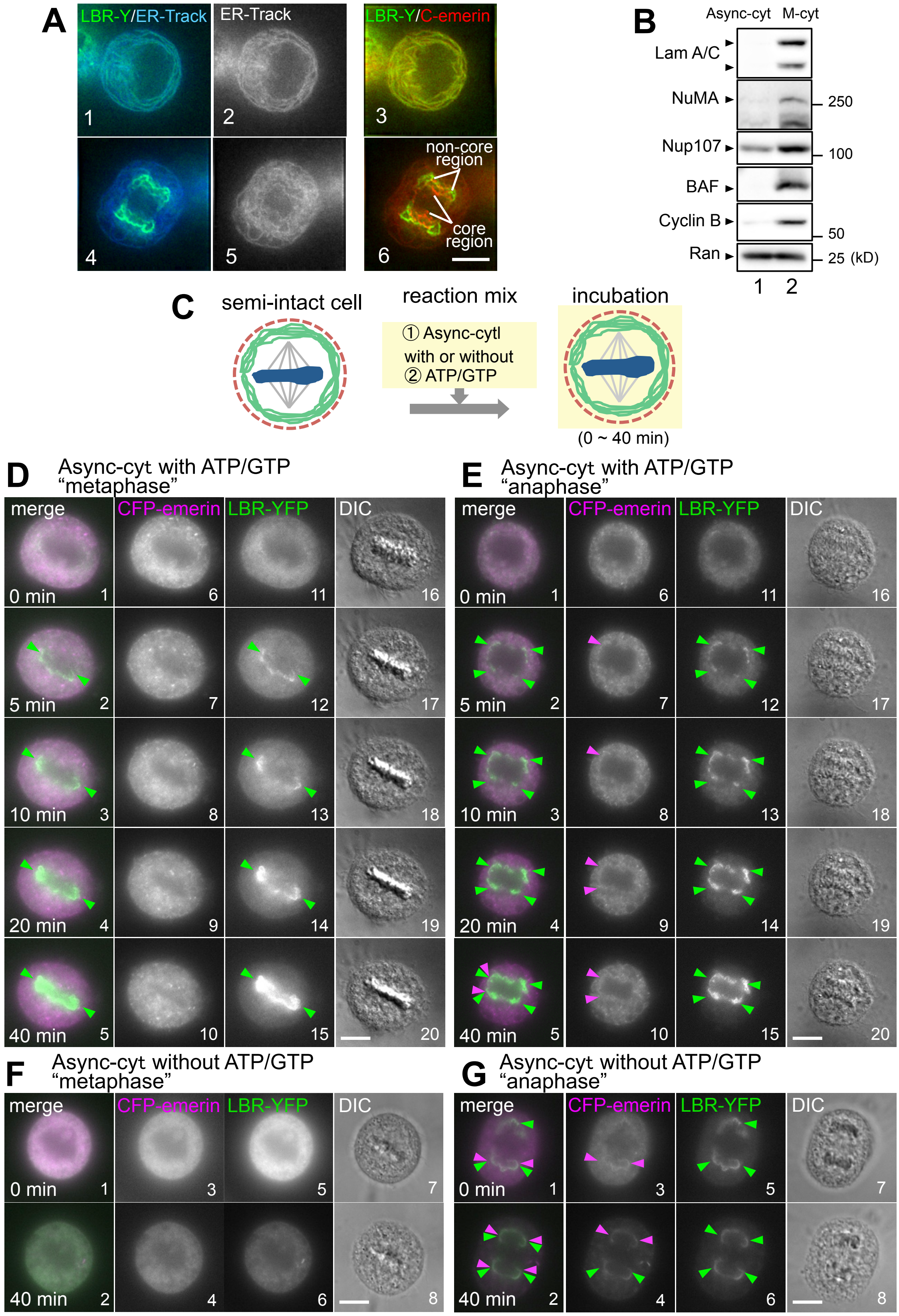

Supplementary figure 2

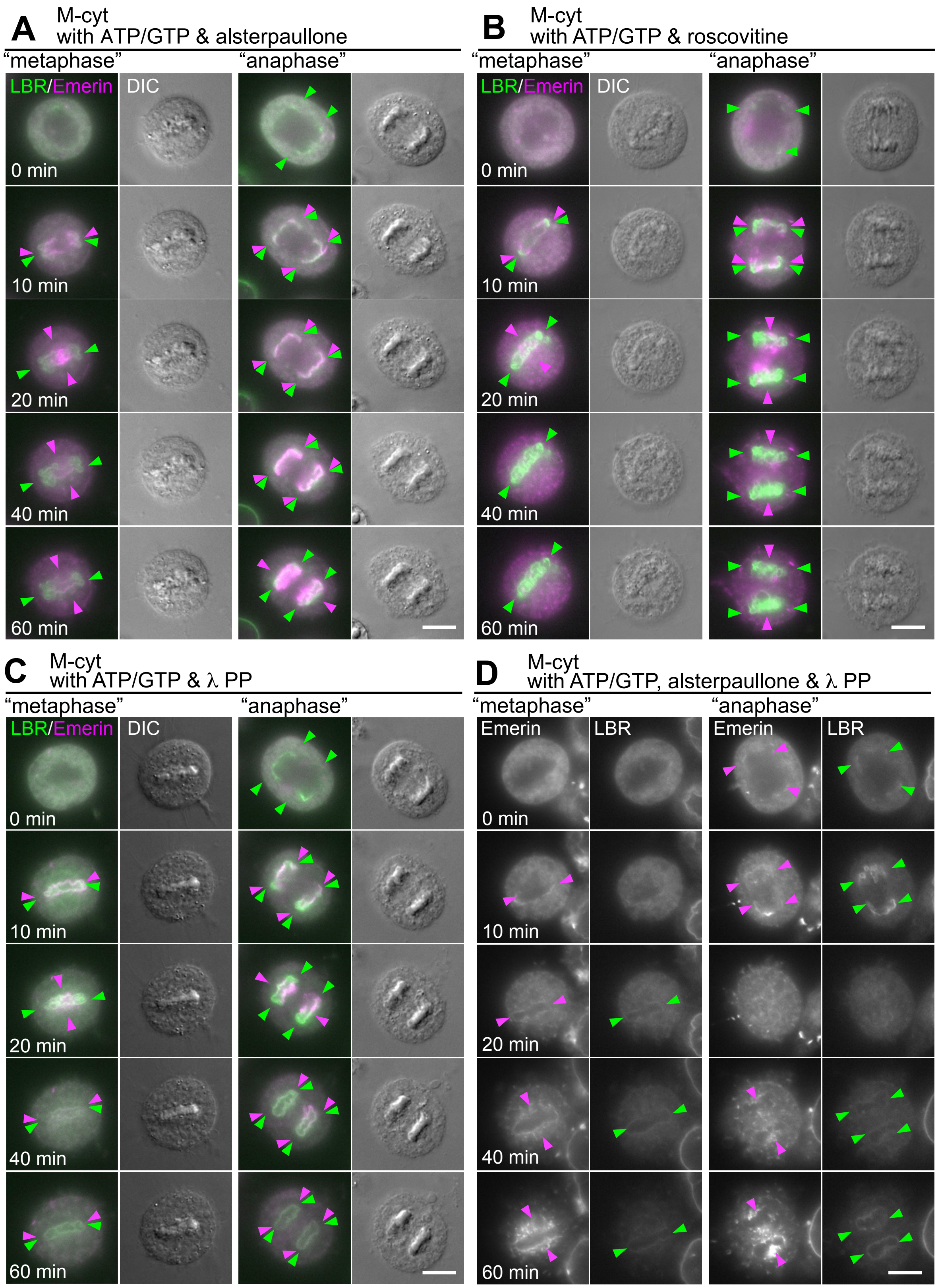

Supplementary figure 3

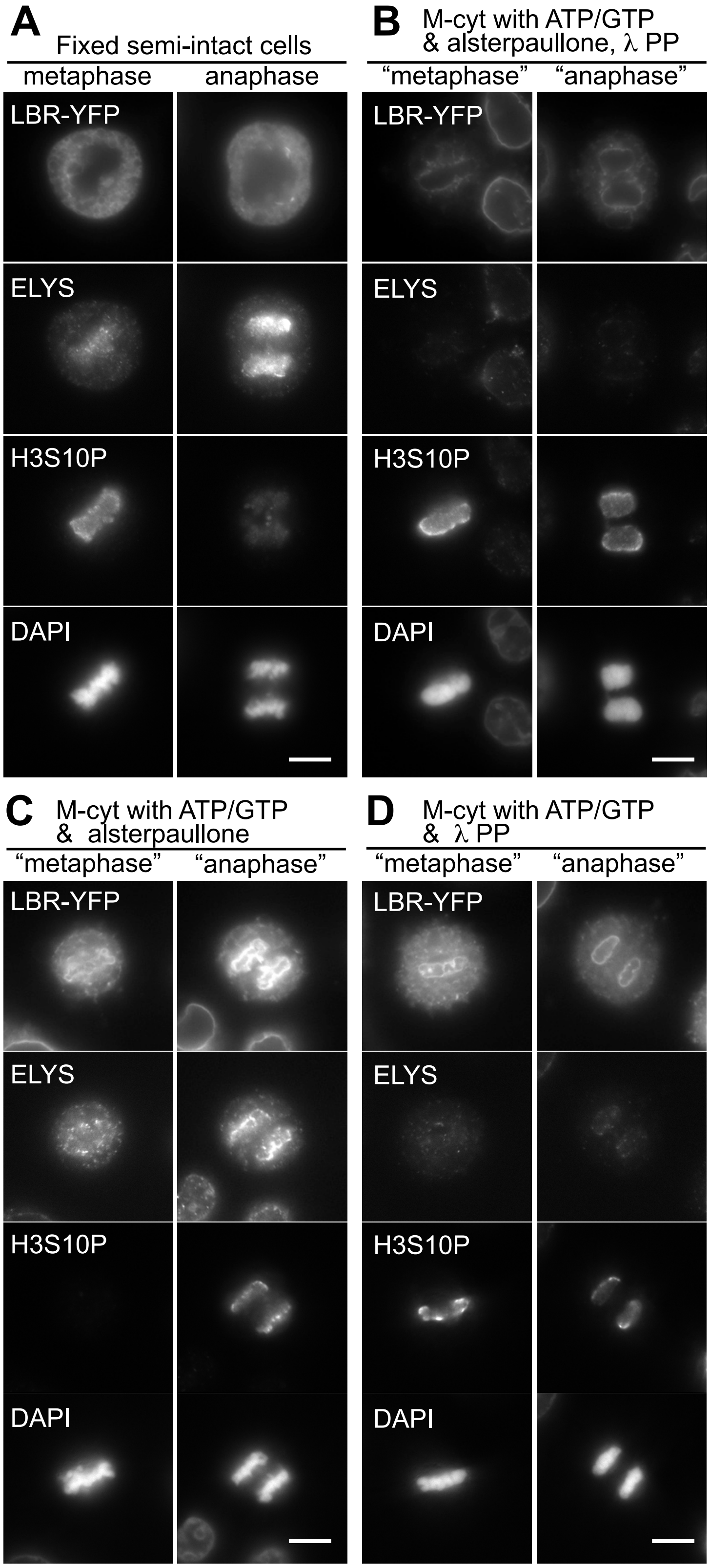

Supplementary figure 4

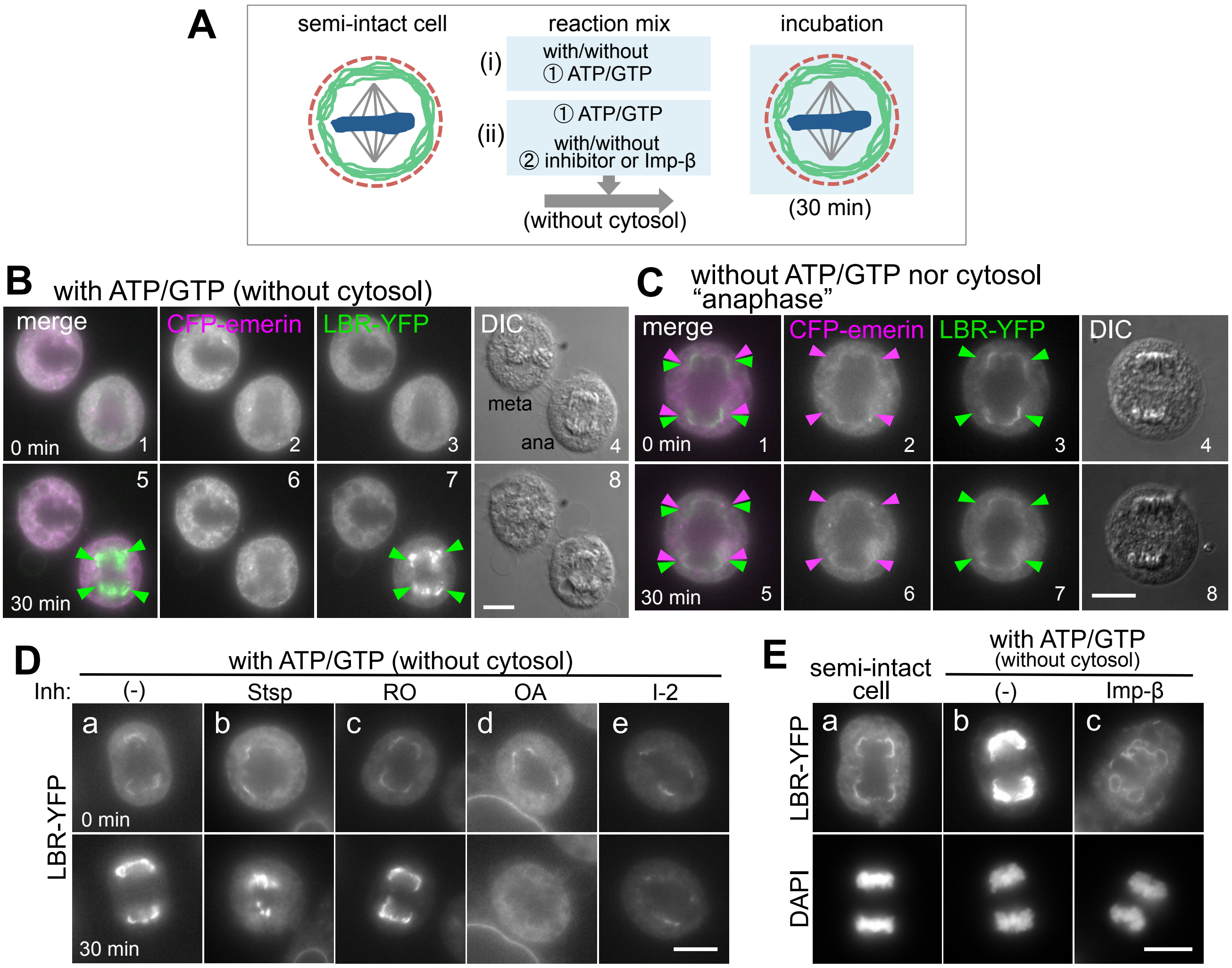

Supplementary figure 5

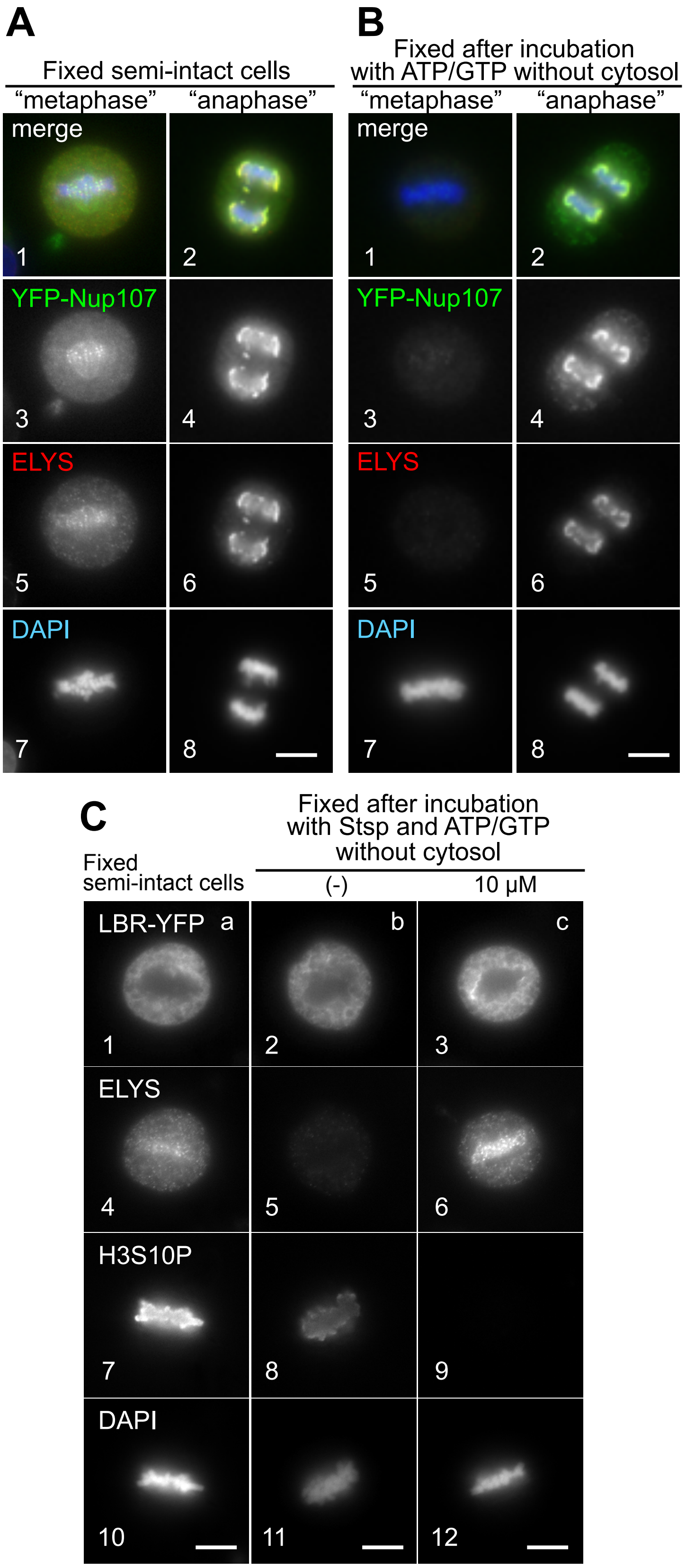

Supplement: Supplementary file 4 — Supplementary Materials [file csf_49_24003_4.zip › 49_24003_4.pdf]
